# Clinical Pharmacology of* Citrus aurantium* and* Citrus sinensis* for the Treatment of Anxiety

**DOI:** 10.1155/2018/3624094

**Published:** 2018-12-02

**Authors:** Carmen Mannucci, Fabrizio Calapai, Luigi Cardia, Giuseppina Inferrera, Giovanni D'Arena, Martina Di Pietro, Michele Navarra, Sebastiano Gangemi, Elvira Ventura Spagnolo, Gioacchino Calapai

**Affiliations:** ^1^Department of Biomedical and Dental Sciences and Morphological and Functional Imaging, University of Messina, Messina, Italy; ^2^Anesthesia, Intensive Care and Pain Therapy, A.O.U. G. Martino Messina, University of Messina, Messina, Italy; ^3^Hospital Pharmacy Service, Policlinico “G. Martino”, University of Messina, Messina, Italy; ^4^Hematology and Stem Cell Transplantation Unit, IRCCS Cancer Referral Center of Basilicata, Rionero in Vulture, Italy; ^5^Department of Chemical, Biological, Pharmaceutical and Environmental Sciences, University of Messina, Messina, Italy; ^6^School and Operative Unit of Allergy and Clinical Immunology, Policlinico “G. Martino”, Department of Clinical and Experimental Medicine, University of Messina, Messina, Italy; ^7^Legal Medicine Section, Department for Health Promotion and Mother-Child Care, University of Palermo, Palermo, Italy

## Abstract

**Objective:**

The aim of this review is to analyze preclinical and clinical studies investigating the anxiety effects of* Citrus aurantium* or* Citrus sinensis* essential oils (EOs).

**Design:**

The bibliographic research was made on the major scientific databases. Analysis included only articles written in English and published on peer-reviewed scientific journals describing preclinical experiments and clinical trials carried out to investigate the antianxiety effects of* Citrus aurantium or Citrus sinensis* EOs on anxiety disorders. Clinical studies reporting the antianxiety effects of products containing* Citrus aurantium *or* Citrus sinensis *EOs in combination with other active substances, including medicinal plants, were excluded. Nine clinical studies fulfilled the criteria adopted for analysis.

**Results:**

Data show that* Citrus aurantium* or* Citrus sinensis *EOs produce anxiolytic effects both in preclinical experiments and in different clinical conditions.* Citrus aurantium* EO aromatherapy reduced anxiety level in the great part of stress conditions studied (subjects affected by chronic myeloid leukemia and preoperative patients) except for a sample of patients subjected to colonoscopy. Exposition to* Citrus sinensis* EO in clinical studies shows to be positive in reducing anxiety level in patients waiting for dental treatment as well as in healthy volunteers submitted to an anxiogenic situation.

**Conclusions:**

Overview of clinical trials conducted with* Citrus aurantium* or* Citrus sinensis* on people with anxiety showed that inhalation or oral administration of* Citrus aurantium* and inhalation of* Citrus sinensis* can exert beneficial effects on anxiety; however, because of incomplete accuracy in the reporting of methodology, further more complete clinical studies are warranted.

## 1. Introduction

Citrus plants derived from the single genus* Citrus* are largely interbreedable. Among the common names given to the various members of the citrus family, orange often refers to the most popular* Citrus sinensis* and* Citrus aurantium* [1]. Chemical composition of* Citrus* plants is characterized by the presence of several polyphenolic classes, including flavones, flavanones, flavonols, flavans, and anthocyanins [2, 3].

Experimental evidences highlight their pharmacological effects including antioxidant, cardioprotective, anti-proliferative, anticancer, and hypolipidemic activities [4–8].

In folk medicine, products derived from the peel and/or whole dried immature fruit of orange plants have been used to treat several health problems such as gastrointestinal disturbances, respiratory disorders as agent for cough [9–12], insomnia, stress disorders, epilepsy, and anxiety [12, 13].

Other citrus species such as* Citrus bergamia* have been described for their effects against stress, psoriasis, and hyperlipidemia [14]. The present article focuses on antianxiety preclinical and clinical effects of the two most common citrus species* Citrus aurantium *and* Citrus sinensis*.

*Citrus aurantium* L., also called Seville orange, sour orange, or bitter orange, is a small citrus tree, about five meters tall, with scented white flowers, belonging to Rutaceae family, originating in eastern Africa, Arabia, and Syria, and cultivated in Spain, Italy, and North America [1, 15].

*Citrus aurantium* is called with several local common names in different countries where it is used for food, fragrance, and medical application. Fruit, peel, leaves, flowers, seeds, and essential oil (EO) of* Citrus aurantium* are used in perfumes and cosmetics, as well as in the food and confectionery industry [16]. Bitter orange oil, obtained from the pressure of the fresh peels, is widely used as a flavoring agent in the food industry and for beverages, particularly liqueurs and soft drinks [17]. The composition of the volatile oils is significantly different in flowers, leaves, and peel. Linalyl acetate (50%) is the main constituent in oil from the leaves (petit grain), and linalool (35%) in oil is derived from the flowers (neroli) [18–20]. Flavones, alkaloids such as synephrine and octopamine, carotenoids, and N-methyltyramine are contained in peel, besides the volatile oil. The main active ingredient in bitter orange extract is the phenyl-ethylamine protoalkaloid p-synephrine which represents about 90% or more of the total protoalkaloids. Fruit peel contains a volatile oil composed of d-limonene, d-linalool, N-acetyl octopamine, gamma-aminobutyric acid, flavonoids, coumarins, triterpenes, vitamin C, carotene, and pectin [21]. Other minor protoalkaloidal constituents in* Citrus aurantium* octopamine, hordenine, tyramine, and N‐methyltyramine are absent or in trace amounts in bitter orange extracts [22–25].

Standardized aqueous-alcoholic extracts of the immature fruits of* Citrus aurantium* are widely consumed in dietary supplements for appetite control, weight management, sports performance, and energy, and bitter orange products are also consumed in the form of food as juices and marmalades [16, 26].

*Citrus aurantium* EO, also known as neroli oil, is widely used in aromatherapy. It has been suggested that it stimulates central nervous system, lowers blood pressure, and has sedative, analgesic, anti-inflammatory, antispasmodic, carminative, digestive, and diuretic effects [27]. It is a strongly scented bitter liquid, produced by hydrodistillation of* Citrus aurantium* fresh lives [28].

*Citrus sinensis* L., named orange or sweet orange, is a millennial small tree belonging to the Rutaceae (citrus) family originated in southern China. The orange tree is small, spiny tree, typically growing to 7.5 m, but occasionally reaching heights up to 15 m, generally with a compact crown. Orange tree grows in tropical, semitropical, and warm temperate regions, becoming the most widely cultivated fruit tree in the world [29, 30]. Orange is the world's most popular fruit and is eaten fresh or drunk as juice. Juice can be consumed directly or further processed into concentrate, and both derivatives are used in soda and cocktail drinks, punches, and liqueurs. Orange fruits and peels are also used in desserts, jams and marmalades, and candied peels, as well as cookies, cakes, and candies. EO derived from orange peels, flowers, leaves, and twigs is used in perfumes; orange seed oil may also be used in cooking or as a component in plastic industry [30].

The sweet orange tree is found more or less in the same places as the bitter orange tree. Sweet orange oil is extracted from the fruit of the tree via cold pressure; it is also possible to distill sweet orange oil [31].

*Citrus sinensis* contains several active secondary metabolites contributing to the pharmacological activities of the plant. In* Citrus sinensis* fruits, peel, leaves, juice, and roots, several types of chemical compounds including flavonoids [2, 32], hydroxyamides, steroids, alkanes and fatty acids, coumarins, carbohydrates, peptides, carbamates and alkylamines, carotenoids, volatile compounds, and minerals such as potassium, magnesium, calcium, and sodium have been identified [12, 33].

*C. sinensis* is a rich source of vitamin C, a natural antioxidant that support the immune system activity [33, 34].* C. sinensis* has been used traditionally, to treat intestinal disorders (such as cramps, constipation, colic, and diarrhea), respiratory disorders (such as cough, cold, bronchitis, and tuberculosis), obesity, menstrual disorder, cardiovascular disease (angina, hypertension), anxiety, depression, and stress [35].

Anxiety disorders are among the leading prevalent causes of global mental disorders [36, 37]. They contribute to favour poor compliance with therapy [38] and insufficient patient adoption of healthy behaviors [39]. On the basis of their negative effects on the results of therapy, it is necessary to find effective interventions.

It is known that inhalation of volatile components of* Citrus* EO is able to influence the activity of brain areas such as the hypothalamus, hippocampus, and pyriform; preclinical and clinical research showed that citrus fragrance can restore stress-induced cortex [40, 41] and immunosuppression [42] and may have potential antidepressant effects in rats [43, 44].

In light of the abovementioned findings, we tried to assess if the potential health effects of both* Citrus aurantium* and* Citrus sinensis* are really effective in the treatment of anxiety conditions. With this aim, we summarized the published reports of preclinical and clinical studies regarding the use of* Citrus aurantium- or Citrus sinensis*-based products in conditions related to anxiety disorders.

## 2. Methods

### 2.1. Research Method and Inclusion Criteria of Clinical Trials

A bibliographic research was carried out independently by two researchers (blinded to the authors and initially on results) in the major scientific databases and search engines of peer-reviewed literature from 2000 to July 2018, on life sciences and biomedical topics (PubMed, Scopus, Embase, Web of Science, and Google Scholar). The following keywords or combination of keywords “Citrus anxiety”, “*Citrus aurantium* axiety”, “*Citrus sinensis anxiety”*, and “clinical trials” were used. Analysis included only articles written in English and published on peer-reviewed scientific journals describing clinical trials and applications of* Citrus aurantium* or* Citrus sinensis* EOs. Articles describing the effects of products containing* Citrus aurantium* or* Citrus sinensis* EOs in combination with other active substances including medicinal plants derivatives were also excluded. The selection and the review of clinical studies written in English regarding* Citrus aurantium* or* Citrus sinensis* EOs were performed in accordance with the Preferred Reporting Items for Systematic Reviews and Meta-Analyses (PRISMA) statement [45]. Methodological quality was assessed using validated tools such as the Consort Statement in Reporting Clinical Trials with Herbal Medicine Intervention (Section 4) [46] and the Jadad Scale [47]. From the eligible articles, two investigators independently extracted data by using a standard data extraction form. Data were considered for therapeutic indication, design of the study, number, sex, and age of subjects, endpoints, adverse effects, and outcome. In all the studies, reporting of adverse reactions was monitored. The absence of adverse reactions was defined as “not reported.” All the authors reviewed all the eligible articles and resolved by discussion any uncertainty regarding the statistical method used to handle the missing data.

## 3. Results

A collection of 94 scientific articles was selected from our bibliography research. Only 17 articles describing effects of* Citrus aurantium *or* Citrus sinensis* treatment for anxiety were corresponding to the inclusion criteria. 63 articles were excluded for title and abstract and 4 were excluded because the products object of the studies were combinations including* Citrus aurantium*.

**Figure 1:**
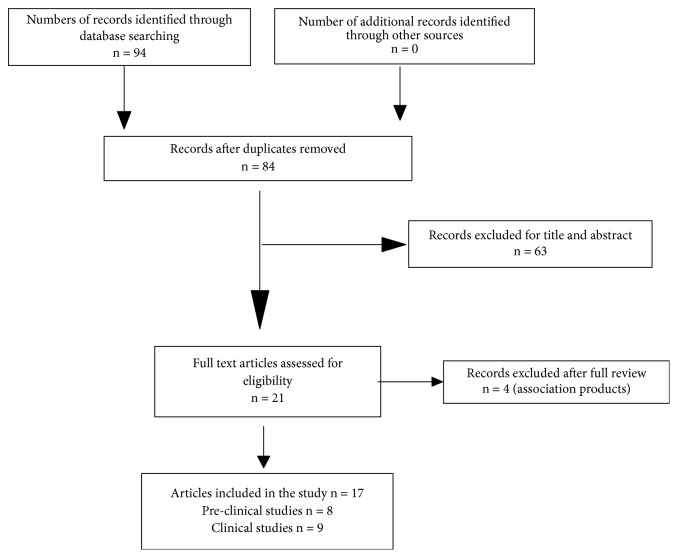
PRISMA flowchart showing the process of literature search and studies selection.

Nine clinical studies were included in the review (Figure 1). In eight clinical studies,* Citrus aurantium *or* Citrus sinensis* were administered for inhalation as aromatherapy, and in one study* Citrus aurantium* was orally administered.

Table 1 summarizes for each preclinical study the authors, the route of administration, animal species, experimental model to study anxiety, dose, and observed effects of* Citrus aurantium *or* Citrus sinensis *administration. Tables 2 and 3 summarize authorship of the paper, therapeutic indication, study design, subjects involved, endpoints, adverse effects, and outcome of all the clinical trials regarding use of* Citrus aurantium *or* Citrus sinensis*, respectively. Tables 4 and 5 report CONSORT items for trials with herbal medicine interventions applied to clinical studies, for* Citrus aurantium* and* Citrus sinensis*, respectively.  Table 6 reports quality assessment of randomized controlled trials by the Jadad scoring system.

### 3.1. Citrus aurantium (Sour/Bitter Orange) Preclinical and Clinical Antianxiety Effects

Antianxiety effects of* Citrus aurantium* were demonstrated through behavioral experiments carried out with laboratory animals.* C. aurantium* EO oral administration in mice increased exploration of the open arms (time spent) in the elevated plus-maze at a dose that did not impair motor activity observed with open-field and rotarod test, which is indicative of anxiolytic-like effect in male mice [48].

The acute administration induced an anxiolytic-like effect in the light/dark transition tests (increased time spent in the light side, and in the number of transitions) and in marble burying (decreased number of marbles buried) without any motor impairment, while repeated administration showed effects in the marble-burying test only. Repeated diazepam administrations did not increase light/dark transitions. Thus, the results suggest an anxiolytic-like effect following acute and repeated* Citrus aurantium* EO administration [49].

Similar results were obtained in another experiment showing that anxiolytic-like effect of oral administration of* Citrus aurantium* EO was reversed by 5-HT1A antagonist WAY100635 but not by flumazenil, a benzodiazepine antagonist, suggesting serotonergic mediation [44].

In another study, inhalation of* Citrus aurantium *EO increased social interactions (time spent in active social interaction) in rats and increased exploration time in the open arms of the elevated plus-maze, suggesting an anxiolytic-like effect at a dose that did not impair motor activity in the open-field test [50].

Acute intraperitoneal administration of* Citrus aurantium* EO, similar to fluoxetine, increased open arm explorations (percentage of time spent and percentage of entries) in the elevated plus-maze in male mice. The authors suggested that the effect of* Citrus aurantium* L. is linked to serotonergic transmission based on a fluoxetine +* Citrus aurantium* EO interaction. However,* Citrus aurantium* EO did not change the anxiolytic effect of fluoxetine in the elevated plus-maze, suggesting no drug interaction [51].

Khosrovi et al. investigated the effect of intraperitoneal injection of* Citrus aurantium *EO on anxiety and its interaction with GABAergic pathways, evaluating the coadministration effects of* Citrus aurantium* and diazepam. Results showed that* Citrus aurantium* EO increased the open arms exploration (increase of percentage time spent) of male mice submitted to the elevated plus-maze. Although diazepam increased open arms exploration (percentage of time spent and percentage of entries), coadministration with* Citrus aurantium* reduced the anxiolytic effect of diazepam. Flumazenil did not alter the effect of* Citrus aurantium*; however, authors suggest that* Citrus aurantium* L. may exert an anxiolytic-like effect acting as partial agonist at the GABA-A receptor/benzodiazepine site [52]. Preclinical antianxiety effects of* Citrus aurantium* are summarized in Table 1.

Complex six studies investigating* Citrus aurantium* EO effects on anxiety levels during different medical conditions were found. All of the six studies in which* Citrus aurantium* was investigated were randomized/controlled clinical trials.

One randomized clinical trial described the effects of* Citrus aurantium *EO in reducing anxiety during labor in a group of Iranian pregnant women [53].

Before the aromatherapy, both groups had the same levels of anxiety; the levels of anxiety evaluated at dilations of 3-4 and 6-8 cm were significantly lower in the aromatherapy group with* Citrus aurantium* compared with the control group, thus suggesting that aromatherapy with* Citrus aurantium *EO could reduce anxiety during labor [53].

A trial was carried out on patients proposed for colonoscopy and divided into two groups. Aromatherapy was performed by inhalation of Sunflower oil (control group) and Neroli oil (experimental group). Results showed that there was no significance difference of procedural anxiety measured by State Trait Anxiety Inventory state (STAI-S) score and procedural pain evaluated by visual analogue scale (VAS), before and after aromatherapy in patients subjected to colonoscopy. Significant lower pre- and postprocedural systolic blood pressure in Neroli group than control group were observed. The authors concluded that the inhalation aromatic agent had an effect on lowering procedural anxiety-related (excessive fear of medical, dental, or surgical procedures that results in acute distress or interference) blood pressure [54].

Patients with a mandibular third molar with B II classification of impacted teeth and American Society of Anaesthesiologists (ASA) class I patients (healthy subjects with no organic pathology), with moderate and high anxiety levels measured through the dental anxiety scale (DAS) questionnaire, were included in another randomized and controlled study.

The ASA clinical status classification system assessed the fitness of patients before surgery. The outcome variables were physiologic measures related to anxiety, including mean blood pressure, respiratory rate, and pulse rate.

After aromatherapy, mean blood pressure, pulse rate, and respiratory rate were significantly lower in the fragrance group during surgery (from the time of sitting in the dental chair to the end of surgery). Vital signs measurements showed significant differences between the two groups, which may be due to the sedative effect of the orange fragrance during surgery. Results suggest that ambient orange fragrance could be helpful in reducing dental anxiety during dental surgical removal [55].

Another study was carried out on a Brazilian volunteers' cohort with chronic myeloid leukemia (CML), treated with standard therapy. The study compared* Citrus aurantium* EO with diazepam anxiolytic effects, in the moment that precedes the collection of medullary material in patients with CML. Systolic and diastolic blood pressure and cardiac and respiratory frequencies were measured.* C. aurantium* EO decreased both systolic and diastolic blood pressure while with diazepam, only systolic blood pressure decreased. The* Citrus aurantium* EO did not decrease the respiratory frequencies.

The use of STAI-S revealed an anxiolytic effect of* Citrus aurantium* EO group of patients with CML but not in the CML diazepam and placebo groups. In conclusion, study results showed an anxiolytic effect of* Citrus aurantium *EO in patients with CML with an improvement of psychological and physiologic parameters. For the authors, this effect has a great clinical relevance, because patients with cancer go through various stressful phases during the disease, and the standard therapy significantly contributes to improve the anxiety level and physiological parameters during a procedure that is a cause of distress [13].

Chaves Neto et al. (2017) studied the anxiolytic effects of* Citrus aurantium* EO in patients experiencing crack withdrawal. Based on the fact that individuals who experience crack withdrawal present a high anxiety trait, anxiety status was induced with the Simulated Public Speaking (SPS) method (subject is requested to deliver a speech in front of a video camera with its image being displayed on a TV screen). Anxiety levels were assessed by the Inventário de Ansiedade Traço-Estado (IDATE), the Brazilian version of STAI-S.

The results demonstrated that subjects in the groups treated with* Citrus aurantium* EO maintained controlled anxiety levels during SPS when compared to the control group (no treatment). Subjects who were administered* Citrus aurantium* EO also maintained states of discomfort and cognitive impairment often associated with anxiety during SPS. However, nebulization of the EO of* Citrus aurantium *provided an acute anxiolytic effect in crack cocaine users exposed to SPS.

Authors' conclusions were that* Citrus aurantium* EO, administered by nebulization, produced anxiolytic effects in crack users in abstinence thus indicating the possibility of* Citrus aurantium* EO use as an alternative complementary therapy in the control of anxiety in users who are abandoning drugs abuse [56].

In a randomized double-blind design, the effect of oral administration of* Citrus aurantium* blossom distillate (CABd) on preoperative anxiety was evaluated. Preoperative anxiety was assessed using STAIS and Amsterdam Preoperative Anxiety and Information Scale (APAIS). The main finding of this study was the confirmation of the anxiolytic effect obtained with oral administration of blossom distillate* Citrus aurantium*. Both STAI-state and APAIS scores were decreased by CABd. On the other hand, neither STAI-state nor APAIS was changed in the placebo group [57].

Results of the studies using* Citrus aurantium* EO showed that inhalation of the oil produces significant anxiolytic effects. Methods for diffusion of EO used in the studies are by direct inhalation with hand-hold nebulizers generally with doses of EO ranging 10 – 50 mL or by dilution of EO in distilled water and successive diffusion through an electric dispenser or by gauzes impregnated with 4 mL of* Citrus aurantium* EO.

### 3.2. Citrus sinensis (Sweet Orange) Pre-Clinical and Clinical Anti-Anxiety Effects

In comparison to findings published about investigation on* Citrus aurantium*, a reduced number of preclinical and clinical experiments were conducted on* Citrus sinensis*. Inhalation of* Citrus sinensis* EO (sweet orange, containing 97% limonene) in rats submitted to elevated plus-maze followed by the light/dark paradigm produced an increase in open arm exploration (% time spent and % number of entries) in the elevated plus-maze, and of the time spent in the lit chamber of the light/dark test. No effect on motor activity, as measured by the total distance travelled in the elevated plus-maze, was detected [58].

Anxiolytic‐like, sedative, and antidepressant‐like potential effects of inhalation of both* Citrus aurantium* and* Citrus sinensis* EOs were evaluated through behavioral tests and measurement of corticosterone and melatonin plasma levels in mice.

Results of behavioural tests showed an anxiolytic‐like and sedative effect of* Citrus sinensis* EO 10% inhalation, without affecting melatonin and corticosterone physiological levels. Inhalation of 10%* Citrus aurantium* EO did not show neither anxiolytic-like effects nor change in melatonin and corticosterone plasma levels [59].

Three randomized and controlled studies investigating on the effects of* Citrus sinensis* were found. Two studies describe the effects of* Citrus sinensis* EO on anxiety in adults and children, respectively, subjected to dental treatments.

A randomized controlled trial was carried out on adult patients waiting for dental treatment to evaluate the anxiolytic effects of* Citrus sinensis*. Orange odor was diffused in the waiting room of odor group through an electrical dispenser whereas in the control group no odor was diffused. For assessing cognitive functions, patients completed the Wortschatz test (WST) [60]. For assessing trait and state anxiety, patients were given the State-Trait Anxiety Inventory (STAI) [61]. The Mehrdimensionale Befindlichkeitsfragebogen (MDBF) [62] were used for assessment of current mood, alertness, and calmness.

Results of the study showed that exposure to ambient orange odor has a relaxant effect compared to control group. Results reported also a sex difference of* Citrus sinensis* effects, showing that women exposed to sweet orange EO had a lower level of state anxiety, a more positive mood, and a higher level of calmness [63].

The effects of orange odor (*Citrus sinensis*) on child anxiety during dental treatment were also evaluated in another study by using salivary cortisol and pulse rate as index of patients' anxiety.

The results of this study showed that the salivary cortisol level and pulse rate significantly decreased in intervention groups by using aromatherapy with* Citrus sinensis* EO [64]. The potential anxiolytic effect of* Citrus sinensis* EO was evaluated in a double-blind, randomized, placebo-controlled clinical trials carried out on healthy volunteers submitted to an anxiogenic situation. Immediately after inhalation, each subject was submitted to the video-monitored version of the Stroop Color-Word Test (SCWT), a method to induce anxiety. During the text, a board with 100 of the color-naming words blue, yellow, red, green, and violet organized randomly in a 10 · 10 matrix is presented to each participant. The color of each word is different from its own meaning (for example, the word “green” printed in red). The participant has to say quickly (in two minutes) the sequence presented, the color of the ink, but not the colors described by the single words. The whole test is recorded and presented to the subject on a monitor during the test [65].

State-Trait Anxiety Inventory (STAI) and Visual Analogue Mood Scale (VAMS) were used to evaluate psychologic parameters. Physiologic parameters were evaluated before the inhalation period and before, during, and after the SCWT. Results showed dose-dependent anxiolytic properties of sweet orange EO [65].

These three studies indicate that* Citrus sinensis* EO exerts anxiolytic effects in anxiogenic situations. One of the studies show that* Citrus sinensis* EO exerts its anxiolytic effects also in children.

## 4. Discussion

EOs physiological and psychological effects are known in folk medicine and aromatherapy for a long time [14, 66]. Aromatherapists have proposed that* Citrus* fragrances have mood-enhancing properties. These effects were confirmed by successful clinical study carried out with citrus fruits oils on patients affected by stress symptoms or depression [67, 68].

Anxiety disorders are the most common type of psychiatric disorders in the general population [69]. Their treatment is difficult because of the important side effects of the drugs used to improve anxiety symptoms, which generally promote dependence [70]. Moreover, common treatments do not benefit all patients and only few of them have a slight resumption [71]. These conditions justify the increasing interest and search for alternative or complementary procedures aimed at improving anxiety symptoms. One of these is aromatherapy, a procedure that uses EOs by inhalation as a treatment for medical purposes [72].

*Citrus aurantium* and* Citrus sinensis *are rich in flavonoids and polyphenolic compounds with numerous pharmacological properties, such as the inhibition of the oxidation of low-molecular weight proteins and platelet accumulation thus contributing to immune cell stability.

They are also used in treatment of mental disorders, inflammation, viral infections, and allergies [73, 74]. Flavonoids effects in reducing anxiety are due to their action as benzodiazepine receptor agonists [57].

A series of review articles regarding the safety and efficacy of bitter orange has been published [75–78]. However, this is the first analysis of clinical studies using* Citrus aurantium* or* Citrus sinensis* for the treatment of anxiety. We analyzed all clinical studies according to the recommendations described in the checklist developed by the Consolidated Standards of Reporting Trials [46] for the reporting of clinical trials using herbal medicinal products (Tables 4 and 5). The clinical trials included in this review have different levels of methodological accuracy. All of studies were randomized and controlled. Only one study regarding* Citrus aurantium *use was carried out with a double-blind methodology (Table 2). Two of the studies regarding* Citrus sinensis* use were carried out with a double-blind methodology, and only one was performed as a crossover study (Table 3).

Since it is a common source of selection bias, a point of weakness in all the studies is the lack of description of the methods adopted to generate random allocation sequences [78] (Table 6). Moreover, all the studies were poor in number of subjects recruited. In four of them, the sample of people recruited did not exceed 50 patients, in four studies, the samples exceeded 50 participants, and only in one study the sample was more than 100 patients (Tables 2 and 3).

All the studies reported statistical data analysis but no one reported sample size calculation. Latin binomial name of the plant was always correctly reported, although not all articles provided an exhaustive description of the characteristics of the product. Procedure adopted to obtain the EO and description of the raw material used to produce the herbal preparations were reported only in one study (Tables 4 and 5). The highest standards in methodology for clinical trials in herbal medicine strongly recommend the reporting of the characteristics of the product to facilitate the reproducibility of the studies. The presence of these data are fundamental to establish a link between the putative efficacy and safety to the single product [46].

Only few studies report qualitative testing producing the chemical fingerprint of the herbal products (Table 4). Finally, one study [13] compares the effects of* Citrus aurantium* against diazepam, a well-established drug prescribed for the treatment of anxiety (Table 2).

## 5. Conclusions

Antianxiety effects of* Citrus aurantium* and* Citrus sinensis* EOs were previously demonstrated through behavioral experiments carried out on laboratory animals. Complex clinical studies considered for this overview suggest that* Citrus aurantium* or* Citrus sinensis* EOs, used for anxiolytic therapy of people prevalently in conditions in which stress is dominating, produce positive effects against anxiety. In particular,* Citrus aurantium* EO aromatherapy reduced anxiety level in the great part of stress conditions studied (subjects affected by chronic myeloid leukemia and preoperative patients) except for a sample of patients subjected to colonoscopy. Exposition to* Citrus sinensis* EO in clinical studies is shown to be positive in reducing anxiety level in patients waiting for dental treatment as well as in healthy volunteers submitted to an anxiogenic situation. However, a definitive conclusion needs further studies because of the reduced number of studies and the small number of patients for each study. Regarding data reporting of clinical studies with* Citrus aurantium*, they appear without description of the characteristics of the herbal product and lack qualitative testing. From the methodological point of view, most of the clinical studies show the lack of important items such as treatment allocation conceal and description of double-blind procedure. On the basis of the present overview, we can conclude that the use of* Citrus aurantium* and* Citrus sinensis* EOs could be useful to reduce anxiety caused by clinical stress conditions; however, results are impaired by the poor accuracy and methodology applied in clinical studies.

## Figures and Tables

**Table 1:** Principal characteristics of pre-clinical studies carried out with *Citrus aurantium* or *Citrus sinensis* essential oil.

**Species**	**Authors**	**Preparation**	**Route of administration**	**Species**	**Anxiety Model**	**Dose**	**Observed Effect**
***Citrus aurantium***	Carvalho and Costa, 2002	*Citrus aurantium* essential oil	Gavage oral administration	Male Swiss mice	Pentobarbital Sleeping Time (induced by sodium pentobarbital 40 mg/kg, i.p.) Elevated Plus Maze Test (EPM) Open Field Test Rota-Rod Test Convulsing Tests (induced by subcutaneous injection of pentylenetetrazole – 85 mg/kg).	Animals were orally treated with *Citrus aurantium* essential oil (0.5 or 1.0 g/kg), extract or fractions (HE, HF, DF and AF at 1.0 g/kg) 30 min before the experiments for the evaluation of the sedative/hypnotic activity, anxiolytic activity (elevated plus maze and anticonvulsant activity or by maximal electroshock.	*Citrus aurantium* 1.0 g/kg increased the sleeping time induced by barbiturates and the time spent in the open arms of the EPM. Both doses of preparation used did not promote deficits in general activity or motor coordination. HF and DF fractions (1.0 g/kg) did not interfere in the epileptic seizures but were able to enhance the sleeping time induced by barbiturates.
	Leite et al., 2010	*Citrus aurantium* essential oil	Inhalation	Male Wistar rats	Open-field behavioral test Social interaction test Elevated plus-maze test (EPM)	*Citrus aurantium* essential oil was administered at the concentrations of 1.0%, 2.5% and 5.0%, w/w, for 7 minutes. Control groups: saline, or diazepam 1.5 mg/ kg i.p.	*Citrus aurantium *essential oil at the concentration of 2.5% increased both the time of the animals in the open arms of the EPM and the time of active social interaction in the open-field being longer than that of the diazepam group.
	Costa et al., 2013	*Citrus aurantium* essential oil	Gavage oral administration	Male Swiss mice	Light/Dark Box Test Rotarod Test (RRT) Forced Swim Test (FST)	*Citrus aurantium* essential oil was administered as single dose (5 mg/kg) or 14-day repeated dose (1 mg/kg/day).	*C. aurantium* EO possesses a significant anxiolytic-like activity, and the present results strongly suggest the involvement of 5-HT1A-receptors.
	Pultrini et al., 2006	*Citrus aurantium* essential oil	Gavage oral administration	Male Swiss mice	Light–dark box test Marble-burying test Rotarod test	Citrus aurantium essential oil 0.5 or 1.0 g/kg in a volume of 10 ml/kg.	In light–dark box test, single treatment with essential oil (0.5 or 1.0 g/kg) increased the time spent by mice in the light chamber and the number of transitions between the two compartments. Single and repeated treatments with essential oil (0.5 or 1.0 g/kg) were able to suppress marble-burying behavior. No impairment on rotarod procedure after both single and repeated treatments with essential oil was observed, denoting absence of motor deficit.
	Khosravi et al., 2014	*Citrus aurantium* essential oil	Intraperitoneal injection	Male albino mice	Elevated plus-maze test (EPM)	Intraperitoneal injection of *Citrus aurantium *L. essential oil was administered at different doses (0.5, 2.5, and 5 percent) for 5 days. Diazepam (0.1 mg/kg) was injected on the fifth day, thirty minutes before *Citrus aurantium *L. essential oil administration. Control and sham group received olive oil.	In groups receiving *Citrus aurantium *L. essential oil at doses of 2.5 and 5 %, there was a significant increase in percent of time spent in the open arms. The injection of diazepam alone or with *Citrus aurantium *L. essential oil caused an increase in the number of entries and the percent of time spent in the open arms. The results of this study show that *Citrus aurantium *L. essential oil can reduce anxiety-related behaviors in male mice that may act via GABAergic system.
	Saketi et al., 2014	*Citrus aurantium* essential oil	Intraperitoneal injection	Male albino mice	Elevated plus maze test	*Citrus aurantium *L. essential oil was administered at doses of 0.5, 2.5, and 5 percent for 5 days. In another set of experiments, after intraperitoneal injection of *Citrus aurantium *L. essential oil at doses of 0.5, 2.5, and 5 percent for 5 days, on day 5, 30 minutes before applying essential oil, fluoxetine (2 mg/kg) was injected.	Injection of *Citrus aurantium *L. essential oil, alone or along with fluoxetine, increased the number of entries into the open arms and the time spent in open arms that may act via serotonergic system.

*Citrus sinensis*	Faturi et al., 2010	*Citrus sinensis* essential oil	Inhalation	Male Wistar rats	Elevated plus-maze Light/dark paradigm	*Citrus sinensis* essential Oil was administered at 100, 200 and 400 *μ*l. Control groups were intraperitoneally injected with diazepam (2 mg/kg) or saline, in an injection volume of 10 ml/kg, 30 min before the behavioural tests.	All doses of *Citrus sinensis* oil demonstrated anxiolytic activity in at least one of the tests and, at the highest dose, it presented significant effects in both animal models, as indicated by increased exploration of the open arms of the elevated plus-maze and of the lit chamber of the light/dark.

*Citrus aurantium* or *Citrus sinensis*	Wolffenbüttel et al., 2018	*Citrus aurantium* or *Citrus sinensis* essential oil	Inhalation	Male adult albino mice	Light– dark test Locomotor activity test Tail‐suspension test Melatonin (MEL) and corticosterone (CORT) assay	10% (v/v*) Citrus aurantium* leaves' EO 10% (v/v) of *Citrus sinensis* peel EO was administered. Tween 80 (1% v/v in distilled water) inhaled solution or intraperitoneal injection of diazepam 2.0 mg/kg, or intraperitoneal injection of 20.0 mg/kg imipramine, or intraperitoneal injection of saline solution (0.9% NaCl) was used as control.	Behavioral tests showed that the inhalation of 10% *Citrus sinensis* EO presents an anxiolytic‐like and sedative effect. Vaporization of 10% *Citrus aurantium* EO for 30 min by mice did not produce anxiolytic‐like or sedative effects. Inhalation of *Citrus aurantium *and *Citrus sinensis* EO did not affect MEL and CORT plasma levels in mice.

**Table 2:** Principal characteristics of clinical studies carried out with *Citrus aurantium* essential oil.

**Authors**	**Indication**	**Study ** **design**	**Subjects (number and age)**	**Treatment**	**Principal endpoints**	**Adverse ** **effects**	**Outcome**
Fernandes Pimenta, et al., 2016	Anxiety in patients with chronic myeloid leukemia (CML)	Randomized controlled study	N = 42 of both sexes. Average age: 45 ± 5 years.	Participants were randomly divided into three groups. Group 1 received 10 mg diazepam as oral dose; Group 2 received *C. aurantium* essential oil (EO) 10 mL diffused in the room through an electric dispenser. Group 3 (placebo) was exposed to the vaporization of saline solution. In the last two groups, the exposure lasted 30min.	The evaluation was performed through psychometric scales STAI-S [State-Trait Anxiety Inventory (STAI)] and physiological measurements (blood pressure and cardiac and respiratory frequency).	Not reported.	Inhalation of *C. aurantium* was associated with a decrease in the STAI-S scores, suggesting an anxiolytic effect. In patients exposed to *C. aurantium*EO or with diazepam, there was a decrease in the systolic blood pressure. A change in all the physiological measurements was observed in the group exposed to *C. aurantium*. The results showed that *C. aurantium* exhibits an anxiolytic effect and reduces the signs and symptoms associated with anxiety in patients with CML.

Pei-Hsin et al., 2010	Anxiety, stress and physiological parameters in patients subjected to colonoscopy	Randomized controlled trial.	N = 27 subjects: 13 in control group and 14 in Neroli group. Average age: 52.26 ± 17.79 years.	Aromatherapy was then carried out by inhalation of Sunflower oil (control group) and Neroli oil (experimental group). One drop (50 ml) of Sunflower oil or Neroli oil placed in handhold-nebulizer was supplied for five minutes.	The anxiety index was evaluated by STAI-S before aromatherapy and after colonoscopy; postprocedural pain index was measured by visual analogue scale (VAS). Systolic and diastolic blood pressure, heart rate and respiratory rate were evaluated before and after aromatherapy.	Not reported	There was no significant difference of procedural anxiety by STAI-S score and procedural pain by VAS before or after aromatherapy. The physiological parameters showed a significant lower pre- and postprocedural systolic blood pressure in Neroli group than control group.

Akhlaghi et al., 2011	Anxiety in ASA physical status I (healthy) patients scheduled for lower limb minor operation under general anesthesia.	Randomized controlled double-blind study	60 outpatients, scheduled for elective minor surgery Age range: 15-60 years.	Participants were divided into two groups of 30 receiving oral *Citrus aurantium* blossom distillate (CABd 1 mL.kg-1) or placebo, respectively, two hours before surgery.	Preoperative anxiety was assessed using both State-Trait Anxiety Inventory (STAI state) and Amsterdam Preoperative Anxiety and Information Scale (APAIS). Heart rate and blood pressure were measured two hours before operation just before premedication.	Not observed.	Patients treated with CABd were significantly less anxious than patients of placebo group (p < 0.05).

Hasheminia et al., 2014	Moderate and high anxiety before and during surgical removal of an impacted mandibular third molar.	Randomized controlled clinical trial.	N = 56; Age range: 15-45; mean age fragrance group: 26.4 ± 5.3 years, mean age no fragrance group: 27.5 ± 5.7 years.	Patients were divided into two groups: fragrance group (19 males, 9 females), control group (12 males, 16 females). Patients of the fragrance condition were exposed to 5 drops (0.25 mL) of *C. aurantium* essential oil poured in 5 L of water and diffused using an electrical dispenser. Patients in the control condition were exposed in the same environment to diffusion of water without fragrance. All the patients (control and experimental) waited about 10 min in the waiting room.	The dental anxiety scale (DAS) questionnaire was used to determine the anxiety level of the patients prior to surgery Mean blood pressure, respiratory rate, and pulse rate were also evaluated.	Not reported	Orange fragrance is effective in reducing anxiety linked to surgical removal of impacted mandibular third molar. Mean blood pressure, respiratory rate, and pulse rate, during surgery, were significantly reduced.

Namazi et al., 2014	Anxiety during labor in primiparous pregnant women.	Randomized controlled trial	126 primiparous women divided into two groups: aromatherapy (n = 63) and control (n = 63). Age range: 18-35 years. Mean age: 26.43 ± 3.21 aroma therapy group; 26.60 ± 3.40.	100 mL of the distillate contained 8 mL *C. aurantium *essential oil. Gauzes impregnated with 4 mL of *C. aurantium *distillate and normal saline were attached to the collar of the participants in the aromatherapy and control groups, respectively. The gauzes were changed every 30 minutes.	Intensity of anxiety was measured at baseline and after the intervention at dilations of 3-4 and 6-8 cm. Data were collected using a demographic and obstetric questionnaire, an examination and observation checklist including vital signs, vaginal examination, uterine contractions, and fetal heart rate, and Spielberger state-trait anxiety questionnaire.	Not observed	The levels of anxiety at dilations of 3-4 and 6-8 cm were significantly lower in the aromatherapy group compared with the control group.

Chaves Neto et al., 2017	Anxiolytic effect of *Citrus aurantium L*. in Crack Users subjected to Simulated Public Speaking (SPS).	Randomized controlled clinical trial.	51 volunteers, subdivided into three groups: Control Group: non-crack users who were not internal to the therapeutic communities (n=17) mean age of 28 years (± 2.01); Nonusers EO Group: non-crack users who were not internal to the therapeutic communities (n=17), mean age of 24 years (± 0.7282) Users EO Group, users of crack that were internal to the therapeutic communities (n=17), mean age of 30 years (± 2,125).	*Citrus aurantium* essential oil was administered by nebulization, 2 drops (0.1 mL) in 1.9mL of distilled water solution with an emulsifier (Tween 80 at 12%), for each subject. Control Group experienced received only the distilled water with an emulsifier.	The Simulated Public Speaking (SPS) method was used. Physiological measures were assessed at specific phases during the experiment. Psychological measures of anxiety were assessed using the Trait-State Anxiety Inventory (IDATE) and the Humor Analog Scale (HAS).	Not reported	Nebulization of C*itrus aurantium* L. EO provided an acute anxiolytic effect in crack cocaine users exposed to SPS.

**Table 3:** Principal characteristics of clinical studies carried out with *Citrus sinensis* essential oil.

**Authors**	**Indication**	**Study ** **design**	**Subjects (number and age)**	**Treatment**	**Principal endpoints**	**Adverse ** **effects**	**Outcome**
Lehrner et al., 2000.	Anxiety in patients waiting for dental treatment.	Randomized controlled study	Total number of 72 patients; age range: 22 - 57 years. Mean age Odor group: males 38.2 ± 9.6 years (age range 30-69 years); females 32.5 ± 9.7 years (age range: 21-50 years). Mean age No-odor group: males 31.4 ± 4.2 years (age range: 24-30years); females 34.6 ± 9.7 years (age range: 22-57 years)	Participants were divided into two groups: odor group: 18 men and 17 women. control group: 14 men, 23 women Ambient odor of orange was diffused in the waiting room through an electrical dispenser in the odor group whereas in the control group no odor was released in the air. Every morning and every noon approximately 0.25 ml, corresponding to five drops, of essential oil was applied to the diffuser.	To assess cognitive function, the Wortschatz test (WST) was used. Postprocedural pain index was measured by visual analogue scale (VAS). State of anxiety was evaluated with the State Trait Anxiety Inventory (STAI) state. For assessment of current mood, alertness, and calmness, the Mehrdimensionale Befindlichkeitsfragebogen (MDBF) was used. Mood, alertness, and calmness was evaluated with the five-point Likert scales.	Not reported.	Relaxant effect of ambient orange odor exposure. Women exposed to orange odor had a lower level of state anxiety, a more positive mood, and a higher level of calmness.

Jaafarzadeh et al., 2017	Child anxiety during dental treatment.	Randomized, controlled, blinded, crossover, clinical trial.	30 children (10 boys, 20 girls). Age range: 6-9 years. Mean age: 7.66 ± 0.84 years First group mean age: 7.80 ± 0.86 years (treated). Second group mean age: 7.53 ± 0.83 years (control).	Patients were randomly assigned into two groups according to crossover design. First group: 15 children (9 girls and 6 boys); mean age 7.80 ± 0.86 years, treated in the absence of orange aroma in the first session (control) and under orange aroma in the second one (intervention). Second group: 11 girls and 4 boys; mean age 7.53 ± 0.83 years, treated under orange aroma in the first encounter (intervention) and without odor in the second one (control). 2 ml of orange essence was placed in a dispenser activated for 2 min every 10 min.	Anxiety of children was assessed with salivary cortisol level and pulse rate before and on completion of each of two dental treatment appointments.	Not reported	Statistically significant reduction of salivary cortisol level and pulse rate in aromatherapy group compared to control group.

Costa Goes et al., 2012.	Healthy volunteers submitted to an anxiogenic situation.	Randomized, double-blind, placebo-controlled clinical trial.	40 males healthy graduate student volunteers. Age range: 18-30 years.	The video-monitored Stroop Color-Word Test was used to elicit anxiety in subjects participating in the study immediately after treatment. Participants were divided into five groups treated as follows: Test aroma. The test aroma consisted of essential oil of *C. sinensis* 2.5, 5, or 10 drops (SO_2.5_, SO_5_, SO_10_); control aroma: tea tree essential oil 2.5 drops; nonaromatic control: The nonaromatic control was distilled water, 2.5 drops (H2O).	Psychologic parameters: STAI, Visual Analogue Mood Scale. Physiologic parameters (heart rate and gastrocnemius electromyogram). Psychologic and physiologic parameters were evaluated before the inhalation period and before, during, and after the SCWT.	Not reported	Individuals exposed to the test aroma (2.5 and 10 drops) presented a lack of significant alterations (p> 0.05) in state-anxiety, subjective tension and tranquillity levels throughout the anxiogenic situation, revealing a dose-dependent anxiolytic activity of sweet orange essential oil.

**Table 4:** Section 4 of elaborations of CONSORT items for trials with herbal medicine interventions applied to clinical studies with *Citrus aurantium* herbal preparations.

**Reference**	**Herbal medicinal product name**		**Characteristics of the herbal product**	**Dosage, regimen and quantitative description**		**Qualitative testing**	**Placebo/control group (rationale for control or placebo used)**	**Practitioner**
	Latin name	Brand name		Dose	Duration of use			
Chaves Neto et al., 2017	Yes	Yes	Yes	Yes	Yes	Yes	Yes	Yes

Fernandes Pimenta et al., 2016	Yes	Yes	Yes	Yes	Yes	Yes	Yes	Yes

Namazi et al., 2014	Yes	Yes	No	Yes	Yes	No	Yes	Yes

Hu et al., 2010	No	No	No	Yes	Yes	No	Yes	Yes

Hasheminia et al., 2014	Yes	No	No	Yes	Yes	No	Yes	Yes

Akhlaghi et al., 2011	Yes	Yes	Yes	Yes	Yes	Yes	Yes	Yes

**Table 5:** Section 4 of elaborations of CONSORT items for trials with herbal medicine interventions applied to clinical studies with *Citrussinensis* herbal preparations.

**Reference**	**Herbal medicinal product name**		**Characteristics of the herbal product**	**Dosage, regimen and quantitative description**		**Qualitative testing**	**Placebo/control group (rationale for control or placebo used)**	**Practitioner**
	Latin name	Brand name		Dose	Duration of use			
Jaafarzadeh et al., 2017	Yes	Yes	Yes	Yes	Yes	Yes	Yes	Yes

Costa Goes et al., 2012	Yes	No	Yes	Yes	Yes	Yes	Yes	Yes

Lehrner et al., 2000	Yes	Yes	Yes	Yes	Yes	Yes	Yes	Yes

**Table 6:** Clinical trials quality assessment according to Jadad score.

	Authors	Was the trial described as randomized?	Was the randomization procedure described and appropriate?	Was the trial described as double-blind?	Was the method of double blinding described and appropriate?	Was the number of withdrawals/dropouts in each group mentioned?	Jadad Score
***Citrus aurantium***	Fernandes Pimenta et al., 2016	Yes	Yes	No	No	No	2
	Chaves Neto et al., 2017	Yes	No	No	No	No	1
	Namazi et al., 2014	Yes	Yes	No	No	Yes	3
	Pei-Hsin et al., 2010	Yes	No	No	No	No	0
	Hasheminia et al., 2014	Yes	Yes	No	No	No	2
	Akhlaghi et al., 2011	Yes	Yes	Yes	Yes	Yes	5

***Citrus sinensis***	Lehrner et al., 2000	Yes	No	No	No	No	0
	Jaafarzadeh et al., 2017	Yes	Yes	Yes	No	Yes	3
	Costa Goes et al., 2012	Yes	Yes	Yes	No	Yes	3

The JADAD scoring system was used for the assessment of randomized controlled trials with the following 5 items:

Was the study described as randomized? (Yes = 1 point, No = 0 points);

Was the randomization scheme described and appropriate? (Yes = 1 point, No = -1 point);

Was the study described as double-blind? (Yes = 1 point, No = 0 points);

Was the method of double blinding appropriate? (Yes = 1 point, No = -1 point. If the answer of Item 3 was No, Item 4 is not calculable);

Was there a description of dropouts and withdrawals? (Yes = 1 point, No = 0 points).

## Abbreviations

EO: Essential oil
STAI-S: State Trait Anxiety Inventory score
VAS: Visual Analogue Scale
ASA: American Society of Anaesthesiologists
DAS: Dental anxiety scale
CML: Chronic myeloid leukemia
IDATE: Inventário de Ansiedade Traço-Estado
APAIS: Amsterdam Preoperative Anxiety and Information Scale
CABd: * Citrus aurantium* blossom distillate
WST: Wortschatz test
MDBF: Mehrdimensionale Befindlichkeitsfragebogen
SCWT: Stroop Color-Word Test
VAMS: Visual Analogue Mood Scale.
